# Carriage of *Streptococcus pneumoniae* 3 Years after Start of Vaccination Program, the Netherlands

**DOI:** 10.3201/eid1704101115

**Published:** 2011-04

**Authors:** Judith Spijkerman, Elske J.M. van Gils, Reinier H. Veenhoven, Eelko Hak, F. Yzerman, Arie van der Ende, Alienke J. Wijmenga-Monsuur, Germie P.J.M. van den Dobbelsteen, Elisabeth A.M. Sanders

**Affiliations:** Author affiliations: University Medical Center, Utrecht, the Netherlands (J. Spijkerman, E.J.M. van Gils, E.A.M. Sanders);; Spaarne Hospital, Hoofddorp, the Netherlands (J. Spijkerman, E.J.M. van Gils, R.H. Veenhoven);; University Medical Center, Groningen, the Netherlands (E. Hak);; Regional Laboratory of Public Health, Haarlem, the Netherlands (E.P.F. Yzerman);; Academic Medical Center, Amsterdam, the Netherlands (A. van der Ende);; Netherlands Vaccine Institute, Bilthoven, the Netherlands (A.J. Wijmenga-Monsuur, G.P.J.M. van den Dobbelsteen)

**Keywords:** Streptococcus pneumoniae, nasopharyngeal colonization, heptavalent pneumococcal conjugate vaccine, infectious disease transmission, herd immunity, parents, infants, bacteria, research

## Abstract

To evaluate the effectiveness of the 7-valent pneumococcal conjugate vaccine (PCV7) program, we conducted a cross-sectional observational study on nasopharyngeal carriage of *Streptococcus pneumoniae* 3 years after implementation of the program in the Netherlands. We compared pneumococcal serotypes in 329 prebooster 11-month-old children, 330 fully vaccinated 24-month-old children, and 324 parents with age-matched pre-PCV7 (unvaccinated) controls (ages 12 and 24 months, n = 319 and n = 321, respectively) and 296 of their parents. PCV7 serotype prevalences before and after PCV7 implementation, respectively, were 38% and 8% among 11-month-old children, 36% and 4% among 24-month-old children, and 8% and 1% among parents. Non-PCV7 serotype prevalences were 29% and 39% among 11-month-old children, 30% and 45% among 24-month-old children, and 8% and 15% among parents, respectively; serotypes 11A and 19A were most frequently isolated. PCV7 serotypes were largely replaced by non-PCV7 serotypes. Disappearance of PCV7 serotypes in parents suggests strong transmission reduction through vaccination.

*Streptococcus pneumoniae* (pneumococcus) is a major cause of respiratory and invasive disease worldwide, particularly in children <5 years of age and elderly persons (*1*). All pneumococcal disease is preceded by nasopharyngeal colonization (*2*). To date, slightly >90 serotypes have been identified. Young children, among whom nasopharyngeal carriage rates are highest, are the main reservoir for pneumococcal spread in families and the community (*3*).

In the United States and other industrialized countries, widespread use of the 7-valent pneumococcal conjugate vaccine (PCV7) (Prevenar; Pfizer, New York, NY, USA) for children has led to a dramatic decline in PCV7-serotype invasive pneumococcal disease (IPD), not only in vaccinated children (*4*) but also in unvaccinated persons of all ages (*5*,*6*). This indirect effect has substantially contributed to favorable cost-effectiveness estimates of the vaccination program (*7*,*8*). However, shifts toward nasopharyngeal carriage of non-PCV7 serotypes may eventually counterbalance the direct and indirect benefits of the vaccine, assuming that non-PCV7 serotypes will display similar disease potential (*9*–*12*). Early evaluation of the effect of pneumococcal vaccination on serotype distribution in disease is hampered by the relative infrequency of IPD and the difficulty of identifying causative agents in respiratory diseases such as pneumonia or in otitis media. Therefore, surveillance of nasopharyngeal carriage of pneumococci in vaccinated and in unvaccinated persons provides another useful tool for monitoring how vaccination affects circulating pneumococcal serotypes.

In the Netherlands, as part of the national immunization program (NIP), vaccination with PCV7 was introduced for all infants born after March 31, 2006, in a 3+1 schedule of vaccinations at 2, 3, 4, and 11 months with no catch-up campaign. To evaluate how this PCV7 vaccination program affected prevalence of pneumococcal serotypes after 3 years, we conducted a cross-sectional observational study of nasopharyngeal carriage of pneumococci, and we compared data from vaccinated children and their parents with data from age-matched pre-PCV7 (unvaccinated) controls.

## Methods

### Study Design

In 2009, after PCV7 vaccination had been conducted for 3 years, we examined nasopharyngeal swabs for pneumococcal carriage from 2 age cohorts: 1) healthy 11-month-old children who had received 3 primary vaccinations according to the Dutch NIP but had not yet received the booster dose at 11 months of age or had had the booster dose within the week before sampling, 2) healthy 24-month-old children vaccinated according to the Dutch NIP. We also examined swabs from 1 parent each for the 24-month-old children. Exclusion criteria for children were known or suspected immunodeficiency, craniofacial or chromosomal abnormalities, coagulation disorders, use of anticoagulant medication, and having older siblings in the household who had received a pneumococcal conjugate vaccine. Parents were excluded if they had a bleeding disorder or used anticoagulant medication. The study (NL24116.000.08) was approved by an acknowledged national ethics committee in the Netherlands. The study was conducted in accordance with the European Statements for Good Clinical Practice.

Data from the vaccinated children cohorts were compared with data from pre-PCV7 control children and their parents derived from a longitudinal, randomized, controlled trial (NCT00189020) that had started in the Netherlands well before national PCV7 implementation for infants. In that trial, children had been included at the age of 6 weeks from July 2005 through February 2006 and were followed-up until 24 months of age. Nasopharyngeal swabs were obtained from children at 12 and 24 months of age and from 1 parent of each of the 24-month-old children; results have been described (*13*).

### Nasopharyngeal Swabs

Trained study personnel collected the nasopharyngeal swabs from children and parents by using a flexible, sterile, dry cotton-wool swab transnasally, according to World Health Organization standard procedures (*14*). Also, a transoral nasopharyngeal swab was collected from parents because the pneumococcal yield is known to be higher for adults when both areas are swabbed (*15*). Transoral swabs of the directly observed posterior pharynx were collected on a rigid cotton–wool swab. All swabs in both studies were processed according to the same study procedures by the same laboratory for microbiology as described (*13*). Briefly, swabs were cultured for *S. pneumoniae*, then 1 pneumococcal colony per plate was subcultured and serotyped by the capsular swelling method (Quellung reaction). All serotype 6A isolates were submitted to the National Reference Laboratory of Bacterial Meningitis (Academic Medical Center, Amsterdam) for further discrimination between 6A and the newly discovered serotype 6C by PCR with primers 5106 and 1301 and primers 6C-fwd and 6C-rev (*16*). Results were confirmed by the Quellung reaction with newly available antiserum to identify 6C serotype (Statens Serum Institute, Copenhagen, Denmark). In a post hoc analysis, all serotype 19A isolates from children were examined by the disk-diffusion method for susceptibility to azithromycin, erythromycin, and penicillin and were further tested by Etest (PDM Epsilometer; AB Biodisk, Solna, Sweden) and classified according to Clinical and Laboratory Standards Institute (M100-S20). Because oral antimicrobial drugs are the driving force for resistance in the community, susceptibility to penicillin was further classified according to breakpoints defined by the Clinical and Laboratory Standards Institute; isolates were considered penicillin susceptible (MIC <0.06 μg/mL), penicillin intermediately resistant (MIC 0.12–1.0 μg/mL), or penicillin resistant (MIC >2.0 μg/mL) (*17*).

### Covariates

A questionnaire, completed by each participant at the time of nasopharyngeal sampling, was used to determine risk factors for nasopharyngeal carriage of pneumococci. The questionnaire asked about the following: age, sex, month of sampling, presence of siblings in the household, daycare attendance, passive smoke exposure indoors, clinical signs of a respiratory tract infection at the time of sampling, antimicrobial drug use within 1 month before the sample was taken, and active smoking of the participating parent.

### Statistical Analyses

Data on carriage of *S. pneumoniae* were compared with data from the pre-PCV7 control cohort at age 12 months (n = 319) and 24 months (n = 321) and the parents of the 24-month-old children (n = 296) in which children were enrolled as described previously (*13*). According to protocol, the primary study outcome was defined as the prevalence of any of the PCV7 serotypes (4, 6B, 9V, 14, 18C, 19F, and 23F) and any of the non-PCV7 serotypes (all other pneumococcus serotypes including nontypeable isolates) in children. On the basis of previous trial results (*13*), we expected that the smallest difference we would detect would be prevalence of non-PCV7 serotypes at age 11 and 24 months with a 29% carriage rate in the pre-PCV7 control cohort and an estimated 40% in vaccinated children. Therefore, before conducting the study, we estimated that a minimum sample size of ≈330 children in each group was needed to detect this difference with 80% power with a 2-sided α of 0.05. Differences in prevalence rates were statistically tested by using a 2-sided χ^2^ or Fisher exact test, where appropriate. Multivariate analysis with binary logistic regression modeling was used to obtain adjusted estimates of the association between the outcomes and intervention as given by adjusted odds ratios (ORs) and their corresponding 95% confidence intervals (CIs).

## Results

Three years after PCV7 implementation in the NIP for all newborns, parents of 11,005 children were invited to participate. A total of 1,045 (9.5%) parents were interested in participating, among which 892 families were assessed for eligibility; 153 families were not assessed because the enrollment target had already been achieved. Of the 892 families, 233 were excluded. The most frequent reason for exclusion was household presence of older siblings who had received a pneumococcal conjugate vaccine (78%). In total, 329 children 11 months of age and 330 children 24 months of age and 1 parent for each of the 24-month-old children (n = 324) were enrolled from February 9 through July 9, 2009.

The mean age of vaccinees was slightly lower (4 weeks at 11 months, and 1 week at 24 months of age; both p<0.001) than that of the pre-PCV7 controls. At 11 months, the proportion of siblings <5 years of age and the exposure to smoking indoors was lower for vaccinees (both p<0.001). At 24 months, the proportion of male participants and the proportion of children who had received antimicrobial drugs within 1 month before the swab was taken was higher among vaccinees (both p = 0.03). Also, the months of sampling differed between both studies (p<0.001; Table 1).

**Table 1:** Characteristics of children and their parents before and 3 years after implementation of PCV7 vaccination program, the Netherlands*

Characteristic	11-mo-old children				24-mo-old children				Parents		
	Pre-PCV7, n = 319	Post-PCV7, n = 329†	p value‡		Pre-PCV7, n = 321	Post-PCV7, n = 330	p value‡		Controls, n = 296	Vaccinees, n = 324	p value‡
Male	156 (49)	181 (55)	0.12		155 (48)	187 (57)	0.03		51 (17)	53 (16)	0.76
Mean age (SD)	12.0 mo (0.3)	11.0 mo (0.3)	<0.001§		24.2 mo (0.6)	24.0 mo (0.3)	<0.001§		34.7 y (4.9)	35.1 y (4.4)	0.27§
Presence of siblings <5 y of age	126 (40)	84 (26)	<0.001		127 (40)	135 (41)	0.73		NA	NA	NA
Day care attendance¶	208 (65)	226 (69)	0.35		224 (70)	233 (71)	0.82		NA	NA	NA
Passive smoke exposure#	21 (7)	5 (2)	0.001		26 (8)	16 (5)	0.09		NA	NA	NA
Signs of RTI**	95 (30)	95 (29)	0.80		82 (26)	69 (21)	0.16		NA	NA	NA
Antimicrobial drug use††	20 (6)	24 (7)	0.60		10 (3)	23 (7)	0.03		9 (3)	20 (6)	0.07
Period of sampling											
Oct–Mar	149 (47)	82 (25)	<0.001		156 (48)	86 (26)	<0.001		NA	NA	NA
Apr–Sep	170 (53)	247 (75)	NA		166 (52)	244 (74)	NA		NA	NA	NA
Active smoking	NA	NA	NA		NA	NA	NA		40 (14)	34 (10)	0.25

*Values are no. (%) except as indicated. PCV7, 7-valent pneumococcal conjugate vaccine; RTI, respiratory tract infection; NA, not applicable. †Swabs taken just before booster vaccination at 11 mo of age or within 1 week after booster vaccination. ‡By χ^2^ test or Fisher exact test (2-sided) where appropriate. §By independent-samples *t* test. ¶Defined as >4 h/wk with >1 child from a different household. #Defined as passive tobacco smoke exposure indoors to >1 cigar or cigarette during >5 d/wk. **Defined by evaluation of parents. ††Defined as use of oral or intravenous antibiotics within 1 mo before sample was taken.

### Carriage of *S. pneumoniae* in Children

Prevalence of any of the PCV7 serotypes was 38% among the pre-PCV7 controls compared with 8% among prebooster children at 11 months (OR 0.13, 95% CI 0.08–0.21) and 36% among the pre-PCV7 controls compared with 4% among children 24 months of age (OR 0.08, 95% CI 0.05–0.14) (Table 2). Prevalence rates for individual PCV7 serotypes were significantly lower among vaccinees except for the infrequently carried serotypes 18C (p = 0.45) and 4 (p = 0.49), which were almost absent before and after introduction of PCV7. Among children 24 months of age, serotype 6B had remained in only 2% of all vaccinated children, serotypes 19F and 18C in 1%, and all other PCV7 serotypes had almost disappeared (Table 3).

**Table 2:** Frequencies of nasopharyngeal carriage of *Streptococcus pneumoniae* in children and their parents before and 3 years after implementation of PCV7 vaccination program, the Netherlands*

Participant s	Pre-PCV7, no. (%)	Post-PCV7, no. (%)†	OR‡ (95% CI)	aOR§ (95% CI)
11-mo-old children	n = 319	n = 329		
PCV7	122 (38)	25 (8)	0.13 (0.08–0.21)	0.14 (0.09–0.23)
Non-PCV7	92 (29)	129 (39)	1.59 (1.15–2.21)	1.64 (1.15–2.32)
All	214 (67)	154 (47)	0.43 (0.31–0.59)	0.44 (0.31–0.63)
24-mo-old children	n = 321	n = 330		
PCV7	114 (36)	14 (4)	0.08 (0.05–0.14)	0.08 (0.05–0.15)
Non-PCV7	97 (30)	148 (45)	1.88 (1.36–2.59)	2.01 (1.43–2.84)
All	211 (66)	162 (49)	0.50 (0.37–0.69)	0.51 (0.36–0.72)
Parents	n = 296	n = 324		
PCV7	25 (8)	2 (1)	0.07 (0.02–0.29)	0.06 (0.01–0.26)
Non-PCV7	25 (8)	49 (15)	1.93 (1.16–3.22)	1.98 (1.16–3.37)
All	50 (17)	51 (16)	0.92 (0.60–1.41)	0.90 (0.57–1.40)

*PCV7, all serotypes included in 7-valent pneumococcal conjugate vaccine; OR, odds ratio; CI, confidence interval; aOR; adjusted odds ratio; non-PCV7, all other serotypes not included in 7-valent pneumococcal conjugate vaccine; all; all pneumococcal serotypes. †325/329 (99%) swabs are taken just before the booster vaccination at 11 mo of age and 4/329 (1%) children had received a booster vaccination within 1 week before the sample was obtained. ‡All ORs are based on comparison with pre-PCV7 control cohort. §For children, ORs were adjusted by multivariate analysis for sex, month of sampling, presence of siblings in the household, day care attendance, passive smoke exposure indoors, symptoms of a respiratory tract infection during sampling, and antimicrobial drug use within 1 mo before the sample was taken. For parents, ORs were adjusted for sex, months of sampling, antimicrobial drug use within 1 month before the sample was taken, and active smoking.

**Table 3:** Frequencies of nasopharyngeal carriage of individual *Streptococcus pneumoniae* serotypes in children and their parents before and 3 years after implementation of PCV7 vaccination program, the Netherlands*

Serotype	11-mo-old children				24-mo-old children				Parents		
	Pre-PCV7, n = 319	Post-PCV7, n = 329†	p value‡		Pre-PCV7, n = 321	Post-PCV7, n = 330	p value‡		Controls, n = 296	Vaccinees, n = 324	p value‡
PCV7											
19F	36 (11)	5 (2)	<0.001		24 (8)	4 (1)	<0.001		9 (3)	2 (1)	0.02
23F	34 (11)	6 (2)	<0.001		28 (9)	0 (0)	<0.001		2 (1)	0 (0)	0.14
6B	26 (8)	12 (4)	0.02		43 (13)	7 (2)	<0.001		5 (2)	0 (0)	0.02
14	10 (3)	0 (0)	0.001		8 (3)	0 (0)	0.003		6 (2)	0 (0)	0.01
9V	9 (3)	1 (0)	0.01		6 (2)	1 (0)	0.07		1 (0)	0 (0)	0.30
18C	6 (2)	1 (0)	0.07		4 (1)	2 (1)	0.45		0 (0)	0 (0)	NA
4	1(0)	0 (0)	0.49		1 (0)	0 (0)	0.49		2 (1)	0 (0)	0.14
Non-PCV7§											
19A	5 (2)	32 (10)	<0.001		9 (3)	21 (6)	0.03		4 (1)¶	6 (2)	0.62
11A	11 (3)	12 (4)	0.89		10 (3)	22 (7)	0.04		3 (1)	8 (2)	0.17
6A#	19 (6)	11 (3)	0.11		17 (5)	9 (3)	0.09		2 (1)	2 (1)	0.93
15B	3 (1)	10 (3)	0.06		8 (3)	6 (2)	0.55		1 (0)	2 (1)	0.62
15C	4 (1)	4 (1)	0.97		2 (1)	8 (2)	0.06		1 (0)	2 (1)	0.62
6C	5 (2)	8 (2)	0.43		5 (2)	10 (3)	0.21		1 (0)	1 (0)?	0.95
22F	4 (1)	7 (2)	0.39		2 (1)	5 (2)	0.45		0 (0)	2 (1)	0.18
10A	1 (0)	6 (2)	0.12		1 (0)	8 (2)	0.04		1 (0)	2 (1)	0.62
16F	1 (0)	6 (2)	0.12		4 (1)	6 (2)	0.75		3 (1)	1 (0)	0.27
23B	5 (2)	5 (2)	1,00		12 (4)	11 (3)	0.78		0 (0)	4 (1)	0.06
35F	2 (1)	5 (2)	0.45		0 (0)	9 (3)	0.004		0 (0)	3 (1)	0.10
NT	1 (0)	6 (2)	0.12		3 (1)	5 (2)	0.73		1 (0)	4 (1)	0.21
Other	31 (10)	17 (6)	NA		21 (7)	26 (8)	NA		8 (3)	12 (4)	NA

*Values are no. (%) except as indicated. PCV7, all serotypes included in the 7-valent conjugate vaccine; NA, not applicable; non-PCV7, all other serotypes not included in the 7-valent conjugate vaccine; NT, nontypeable. †325/329 (99%) swabs were taken just before the booster vaccination at 11 mo of age, and 4/329 (1%) children had received a booster vaccination within 1 wk before the sample was obtained. ‡All p values are for comparison with control group and calculated with χ^2^ or 2-tailed Fisher exact test where appropriate. §Only non-PCV7 serotypes with >5 isolates in 11- or 24-mo-old children or in parents are included in this table. ¶In only 1 parent, pneumococci were present in both samples but with detection of a different serotype; serotype 19A was found in the transnasal swab and serotype 3 was found in the transoral swab. Serotype 19A is included in this table. #After discrimination between 6A and 6C by PCR, different serotypes were found by PCR compared with Quellung: 3 isolates (serotypes 6B [n = 1] and 14 [n = 2]) in 24-mo-old controls and 2 isolates (serotypes 11 and 15) in 24-mo-old vaccinees. These serotypes were not included in this table.

In contrast, corresponding prevalences of non-PCV7 serotypes were 29% and 39% among children 11 months of age (OR 1.59, 95% CI 1.15–2.21) and 30% and 45% among children 24 months of age (OR 1.88, 95% CI 1.36–2.59) in the pre-PCV7 and post-PCV7 cohorts, respectively (Table 2). In prebooster children, serotype 19A had become the most prevalent (10%), serotype 11A had remained stable at ≈4%, followed by 6A and 15B (each 3%). At 24 months, serotype 11A had become the most prevalent serotype (7%), followed closely by 19A (6%); proportions of each of these serotypes had doubled among the 24-month-old cohort compared with the pre-PCV7 control cohort. Among children 11 and 24 months of age, serotype 6A had declined while serotype 6C had increased, but the numbers were too small for statistical significance (Table 3). The overall prevalence rate for carriage of *S. pneumoniae* among the pre-PCV7 controls was 67% compared with 47% among prebooster children at 11 months of age (OR 0.43, 95% CI 0.31–0.59) and 66% compared with 49% at 24 months of age (OR 0.50, 95% CI 0.37–0.69), respectively (Table 2).

Susceptibility testing of all serotype 19A isolates showed that no isolates were penicillin resistant. Among the pre-PCV7 controls and among vaccinated children, respectively, 0 and 3 (6%) isolates were intermediately resistant to penicillin, and 0 and 2 isolates (4%) were nonsusceptible to azythromycin and to erythromycin.

### Carriage of *S. pneumoniae* in Parents

Prevalence of any of the PCV7 serotypes was 8% among parents of 24-month-old children in the pre-PCV7 controls and 1% among parents of vaccinated children 3 years after PCV7 implementation (OR 0.07, 95% CI 0.02–0.29). Corresponding figures for non-PCV7 serotypes were 8% and 15% (OR 1.93, 95% CI 1.16–3.22), respectively. A stable pneumococcal prevalence rate of 17% and 16% was observed among parents (OR 0.92, 95% CI 0.60–1.41), respectively (Table 2). Before PCV7 implementation, the most frequently found pneumococcal serotypes among parents had been 19F (3%), 14, and 6B (each 2%). After PCV7 implementation, serotypes 11A (2%) and 19A (2%) were the most frequently isolated, and serotype19F was the only PCV7-included serotype recovered, albeit in only 2 of 324 parents (Table 3).

### Multivariate Analysis of Covariates

Unadjusted associations are shown as ORs in children and parents (Table 2). Multivariate analysis showed that associations after adjustments for some potential confounders did not differ from the unadjusted associations.

## Discussion

Three years after NIP implementation of PCV7 for all newborns in the Netherlands, PCV7-serotype carriage of *S. pneumoniae* was reduced 80%–90% among vaccinated children at 11 and 24 months of age. Among parents of vaccinated children, carriage of PCV7 serotypes had almost disappeared. This impressive reduction of PCV7-serotype carriage in infants is larger than that observed in clinical trials, which showed 50%–60% reduction in PCV7 rates after conjugate vaccination, and should be attributed to herd effects (*13*,*18*,*19*). Herd effects would also account for the disappearance of PCV7 serotypes in parents. This large effect might in part be a result of a high pneumococcal vaccine uptake because 94.4% of all 2-year-old children in the Netherlands have been fully vaccinated (*20*). Our data confirm the major role of young infants in the transmission of pneumococci in the community.

Herd effects may also have contributed to the reported unexpectedly high reductions of otitis media (by 43%) (*21*) and all-cause pneumonia (by 33%) (*22*) in young children in the United States since PCV7 introduction. These reductions exceed overall vaccine efficacy found in randomized controlled trials: 6%–9% reduction of otitis media (*9*,*23*), and 4% reduction of all-cause pneumonia (*24*). However, a recent US study on community-acquired pneumonia (with radiographic confirmation) found no consistent reductions in pneumonia rates among children and adults, except for children <1 year of age (*25*). Whether this finding is the result of replacement disease by other nonvaccine pneumococcal serotypes, other pathogens, or other causes remains to be evaluated. Nasopharyngeal serotype replacement remains a potential drawback of vaccination with pneumococcal conjugate vaccines.

Increased rates of carriage of nonvaccine serotypes were also observed in this study. In vaccinated infants and their parents; serotypes 19A and 11A were the most frequently carried serotypes in the Netherlands. In the United States, multidrug resistant serotype 19A has become a frequent cause of IPD as well as of otitis media in children (*26*,*27*). There is ongoing debate about the actual role of PCV7 introduction and the increase in serotype 19A; antimicrobial drug pressure and secular trends have been emphasized (*28*). In our study, however, post hoc susceptibility testing of all 19A isolates showed a low prevalence of nonsusceptible strains among controls and vaccinees. In addition, our group previously reported a significant increase in serotype 19A carriage after PCV7 vaccinations in a study conducted in a randomized controlled setting (*29*), excluding secular trends and indicating a direct role of PCV7. A trend toward lower carriage rates of serotype 6A and higher carriage rates of serotype 6C was observed in both age groups, suggesting PCV7 cross-protection for serotype 6A but not for serotype 6C, in line with other carriage studies (*30*). However, serotypes 11A and 6C have not yet been reported as a frequent cause of IPD in the Netherlands (*12*).

Observed changes in prevalence of serotype carriage may not be entirely random but may be directly related to the serotype capsule size, which in turn is related to the polysaccharide composition and metabolic costs of the capsule for the bacterium (*31*). Pneumococci with larger capsules are more resistant against nonopsonic phagocytosis and more commonly colonize young children. Our results agree with results of Weinberger et al., which show a significant increase in carriage of highly encapsulated serotypes such as 19A, 11A, 10A, and 35F (*31*). Furthermore, the serotype-specific capsule has been shown to be a major factor in the potential to cause IPD, independent of genetic background and temporal or geographic settings (*32*). This serotype-specific difference in disease potential has also been shown for mucosal infections, although the differences between serotypes were less apparent compared with differences in invasive potential (*33*). In addition, serotypes are independently associated with IPD severity (*34*,*35*). Harboe et al. showed that highly encapsulated and frequently carried serotypes such as 11A, 10A, and 19A have high mortality rates among healthy persons >5 years of age that are comparable to PCV7 serotypes such as 19F or 6B. Therefore, replacing serotypes in carriage may potentially cause equally severe disease. Furthermore, frequently carried serotypes are more likely to affect patients with concurrent illnesses than are infrequently carried serotypes with high invasive disease potential such as serotypes 1 and 7F (*36*). Replacement with highly encapsulated pneumococci may therefore substantially reduce vaccine benefits, especially for those who are older or have chronic disease (*37*). However, in addition to the prevalence in carriage and the disease potential of the serotype, the prevalence and severity of pneumococcal disease are also associated with genetic background and presence of drug-resistant clones (*38*) and depends on population or patient characteristics (*36*). As a consequence, it is crucial to monitor and critically evaluate all of these aforementioned aspects.

In contrast to several other carriage studies (*11*), our study found a significant reduction (20% at 11 months and 17% at 24 months of age) in overall pneumococcal carriage 3 years after the PC7 vaccination program began. Previously, we reported a 10% decline in overall pneumococcal carriage after PCV7 vaccination in a randomized controlled trial setting in the Netherlands after reduced-dose schedules and before implementation of PCV7 in the national immunization program for children (*13*). Herd effects may have contributed to the larger reduction in PCV7-serotype and overall pneumococcal carriage in the present surveillance study. We must, however, be cautious about ascribing the reported reduction in overall pneumococcal carriage in children to the introduction of PCV7 because this was an observational study and other unmeasured factors such as viral infections, seasonal variations, and temporal trends could not be taken into account (*39*). Among parents, overall pneumococcal carriage did not change, but low carriage rates in adults make it harder to detect significant changes. Because the same study procedures were followed by the same well-trained research nurse team and laboratory personnel in both studies, we do not think that the observed carriage reduction is an artifact.

For evaluation of our study results, some potential limitations should be taken into account. First, because our data are observational, we showed associations and no causalities between the introduction of PCV7 and changes in pneumococcal colonization. Although several potential confounders were measured and appeared to differ between the comparison groups, multivariate analysis showed that our results were quite robust. Second, the postvaccination data came from a cross-sectional cohort study including 2 separate age groups, whereas the pre-PCV7 control data were derived from a longitudinal study in which data were collected from the same children at age 12 and 24 months. However, we previously found that potential within-person dependency was not substantially affecting these carriage data, probably because of the large interval between carriage sample collections (*13*). Third, the study was not adequately powered to evaluate serotype-specific differences. Lastly, we used a single-colony method for serotyping in both studies. Currently, improved techniques for detection of multiserotype carriage, e.g., the newer, more sensitive PCRs, are available. Multiple serotype carriage methods might have revealed more nonvaccine strains in both studies, pointing to unmasking instead of true replacement after eradication of vaccine strains. The strengths of our study are the relatively high carriage rates found in both studies in the Netherlands compared with other Western countries, a high PCV7 uptake in the NIP, and the possibility to evaluate the effect of vaccination with PCV7 on pneumococcal carriage in adult contacts. Also, drug-resistant clones do not confound the results because antimicrobial drug use and consequent resistance are low in the Netherlands compared with other European countries (*40*).

Since 2009, pneumococcal vaccines with broader coverage have been licensed and will be introduced into vaccination programs worldwide. The effects of these broader coverage vaccines on potential shifts in pneumococcal serotypes in the nasopharynx are still largely unknown. To predict the long-term health and economic effects, close monitoring is warranted.

In conclusion, 3 years of vaccination with PCV7 has led to impressive shifts in serotype-specific carriage of *S. pneumoniae* in children and their parents. This finding indicates a major role of infants in transmission of pneumococci in the population.
